# Vitamin K Contribution to DNA Damage—Advantage or Disadvantage? A Human Health Response

**DOI:** 10.3390/nu14204219

**Published:** 2022-10-11

**Authors:** Julia Kaźmierczak-Barańska, Bolesław T. Karwowski

**Affiliations:** DNA Damage Laboratory of Food Science Department, Faculty of Pharmacy, Medical University of Lodz, ul. Muszynskiego 1, 90-151 Lodz, Poland

**Keywords:** vitamin K, anti-cancer, ROS

## Abstract

Vitamin K is the common name for a group of compounds recognized as essential for blood clotting. The group comprises phylloquinone (K1)—a 2-methyl-3-phytyl-1,4-naphthoquinone; menaquinone (K2, MK)—a group of compounds with an unsaturated side chain in position 3 of a different number of isoprene units and a 1,4-naphthoquinone group and menadione (K3, MD)—a group of synthetic, water-soluble compounds 2-methyl-1,4-naphthoquinone. However, recent epidemiological studies suggest that vitamin K has various benefits that go beyond blood coagulation processes. A dietary intake of K1 is inversely associated with the risk of pancreatic cancer, K2 has the potential to induce a differentiation in leukemia cells or apoptosis of various types of cancer cells, and K3 has a documented anti-cancer effect. A healthy diet rich in fruit and vegetables ensures an optimal supply of K1 and K2, though consumers often prefer supplements. Interestingly, the synthetic form of vitamin K—menadione—appears in the cell during the metabolism of phylloquinone and is a precursor of MK-4, a form of vitamin K2 inaccessible in food. With this in mind, the purpose of this review is to emphasize the importance of vitamin K as a micronutrient, which not only has a beneficial effect on blood clotting and the skeleton, but also reduces the risk of cancer and other pro-inflammatory diseases. A proper diet should be a basic and common preventive procedure, resulting in a healthier society and reduced burden on healthcare systems.

## 1. Introduction

Next year marks the 80th anniversary of the award of the Nobel Prize to two biochemists, Edward A. Doisy and Henrik Dam, in recognition of their research into the chemical nature of vitamin K in 1943. Yet, the role of vitamin K remains underestimated, being associated in most minds almost solely with the problem of blood clotting. Therefore, the present review is intended to provide a fuller overview of the benefits and functions of vitamin K in the human metabolism.

**Figure 1:**
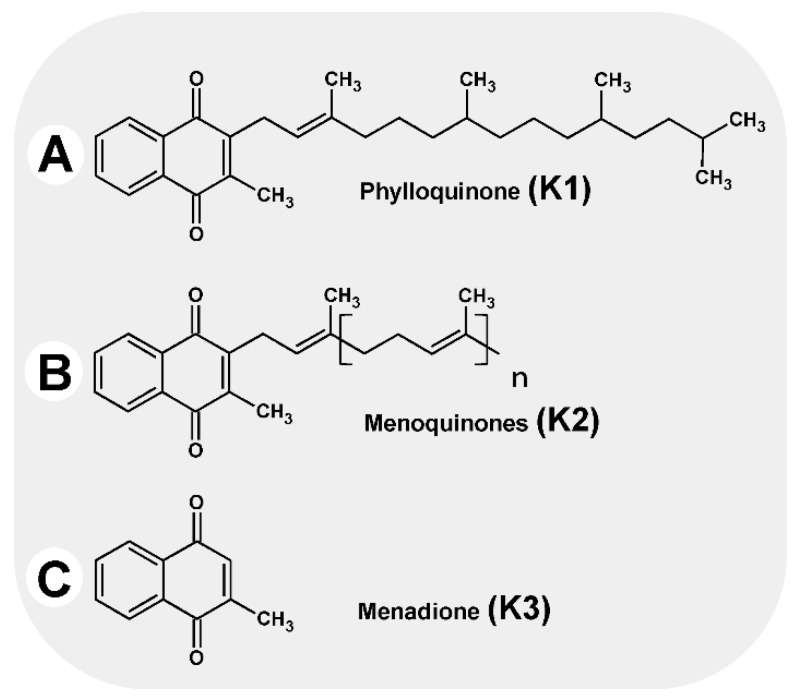
Vitamins K.

Vitamin K is the generic name for the group of fat-soluble compounds with a common chemical structure, sharing a 2-methyl-1,4-naphthoquinone ring with (3C) isoprenoid residues attached to them (Figure 1). In terms of structure, they can be divided into three subgroups as follows [1]:

Vitamin K1 (phylloquinone, phytomenadione), with three saturated and one unsaturated isoprenoid group in a trans-configuration

Vitamin K2 (menaquinone), with between one and 13 unsaturated isoprenoid residues on carbon number three, designated MK-4 to MK-13

Vitamin K3 (menadione), a synthetic derivative with high biological activity; the molecule has no side chain in its structure.

The most common form of vitamin K in foods is phylloquinone (K1) [2,3]. Phylloquinone, closely related to chloroplast membranes, is present in plants where it serves as an electron carrier in photosynthesis and as an electron acceptor to create disulfide bonds [4,5]. The main source is green leafy vegetables rich in chlorophyll, such as spinach, broccoli, Italian cabbage, Brussels sprouts, lettuce, and parsley [6]. Other sources of phylloquinone include margarine and oils (soybean, rapeseed, and olive oil [7]).

Menaquinones (MK), which are mainly bacterial in origin, are found in small amounts in various dairy and fermented products. In the Western diet, the main source of menaquinones (especially MK-7) is cheese, while in Asian cuisine it is natto, produced by fermenting soybeans with *Bacillus subtilis natto* bacteria [7]. Human intestinal microbiota also produce MK-n, although bioavailability is limited as they remain largely bound to the microbial cell membrane [8,9]. MK-4 is unique among menaquinones as it is a product of metabolic conversion from phylloquinone in various tissues in the intestinal mucosa, brain, kidneys, and pancreas [6,10,11].

Menadione, or vitamin K3, is a synthetic form of the vitamin, but it is also formed in the body as a result of the metabolic conversion of phylloquinone. Menadione is converted into MK-4 in the endoplasmic reticulum, with UBIAD1 (UbiA prenyltransferase domain-containing protein 1) as the catalyst [11].

While a good diet and healthy intestines can guarantee a sufficient supply of vitamin K, sadly, the “Western diet”, characterized by excessive consumption of high-energy, refined food, with low levels of vegetables and fruit, and significant levels of antibiotics in meat and other food products, can lead to subclinical vitamin K deficiency.

## 2. Vitamin K in Cell Biology

Vitamin K is responsible for the gamma carboxylation of glutamic acid (Glu) in proteins, resulting in the formation of γ-carboxyglutamic acid (Gla); Gla is capable of affecting protein activation by binding calcium ions. Proteins containing Gla are found in various types of tissues and organs of our body, such as the lungs, pancreas, testes, thyroid, thymus, and kidneys [8].

**Figure 2:**
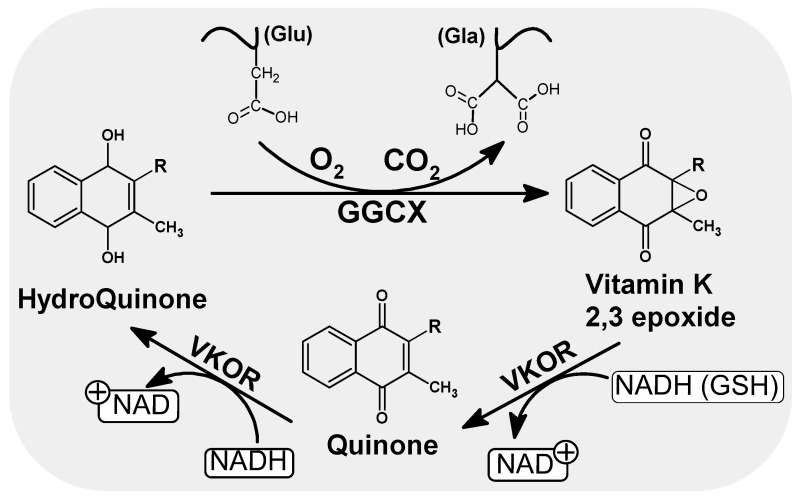
Scheme of the oxidation and reduction of quinones in the cell. As a result of GGCX catalyzed carboxylation in the presence of hydroquinone, CO_2_ and O_2_, the Glu residues of the vitamin K dependent proteins are converted to gammacarboxyl glutamate (Gla). During this conversion, hydroquinone is oxidized to epoxide. Regeneration of the active form of vitamin K (hydroquinone) is handled by VKOR and leads to the consumption of other reduction equivalents such as NADH or/and GSH. NADH/NAD: Nicotinamide adenine dinucleotide (an oxidized and reduced form), GSH: Glutathione, VKOR: Vitamin K epoxide reductase.

Vitamin K undergoes a number of redox transformation processes in various tissues, known as the vitamin K cycle, which allows glutamic acid carboxylation to take place. The cycle involves gamma-glutamyl carboxylase enzymes (GGCX), an integral protein of endoplasmic reticulum (ER) membranes, warfarin-sensitive vitamin K 2,3-epoxide reductase (VKOR) and the reduced form of vitamin K (hydroquinone) (Figure 2). Briefly, hydroquinone (the active form of vitamin K), is transformed into the epoxy form of vitamin K, which is then reduced to quinone by VKOR [12,13,14]. Quinone is then restored to hydroquinone by the influence of reductase in the presence of NADH (Figure 2). This can again participate in the next cycle of reactions. This process of recycling vitamin K means that each molecule can be used up to several thousand times, which explains its low daily requirement compared to other vitamins [15,16].

The best-known vitamin K-dependent proteins are those affecting clotting. However, many other GLA proteins are known, including osteocalcin, matrix Gla protein (Mgp), growth arrest specific protein 6 (Gas6), S protein, transforming growth factor beta inducible protein (TGFβI), and periostin [17,18]. 

This great variety of dependent proteins show that the pleiotropic effects of vitamin K go far beyond ensuring the proper functioning of the clotting processes [19]. There is evidence that vitamin K antagonists (VKA) contribute to cardiovascular damage [20,21] In turn, dietary supplementation with vitamin K is a safe and feasible option for slowing down vascular disease [22]. Recent evidence points to the protective effect of vitamin K on blood vessels, by reducing inflammation and stress ER [23,24]. Similarly, vitamin K has been observed to play an important role in the protection of immature oligodendrocytes or neurons, which are highly sensitive to oxidative damage. Furthermore, evidence shows that vitamin K is involved in preventing arachidonic acid-induced oxidative damage to oligodendrocytes by indirectly blocking 12-lipoxygenase (12-LOX) activation and prevented arachidonic acid-induced accumulation of the 12-HETE pro-oxidative mediator [25]. This is explained by its antioxidant properties: VK acts as a strong free radical scavenger, counteracting the accumulation of intracellular free radicals and inhibits death induced by GSH depletion [25,26,27]. The major intracellular antioxidant that plays a key role in the maintenance of cellular redox homeostasis is reduced glutathione (GSH). GSH depletion leads to oxidative stress as a result of the accumulation of endogenous reactive oxygen species (ROS). In studies of premyelinating oligodendrocytes (OLs) and immature cortical neurons, deprivation of cysteine from the culture medium leads to GSH deprivation and cell death within around 20 h. This effect is prevented by the presence of K1 or MK4 in a concentration of 0.1 µM [26]. The major form present in brain homogenates is MK-4, although its concentration depends on the supply of K1 in the diet. Menaquinone-4 concentration was found to be positively correlated with sulfatide and sphingomyelin content, and regions with high concentration of MK-4 were also the richest in sphingomyelin and sulfatides [28].

A growing body of evidence indicates that vitamin K plays a role in maintaining a healthy skeleton, brain and blood vessels [29,30], and that anticoagulants acting as vitamin K antagonists increase the risk of vascular, skeletal, and neurodegenerative diseases [31,32]. Vitamin K deficiency is associated with a higher risk of chronic diseases involving chronic inflammation. Studies in human cell lines and animals indicate that vitamin K inhibits inflammation in animals [33]. 

DNA microarray analysis indicated that the liver of K-deficient rats demonstrated elevated expression of the genes involved in the acute inflammatory response (1-acid glycoprotein, fibrinogen chain, 2-macroglobulin, and metallothionein-2). Moreover, supplementation with vitamin K1 inhibited lipopolysaccharide (LPS)-induced inflammation; the authors attribute this inhibition to vitamin K-dependent activated protein C (APC). In addition to this anticoagulant effect, APC also has anti-inflammatory, antiapoptotic, and gene modulatory effects [34]. Its anti-inflammatory effect is realized by inhibiting the release of cytokines, e.g., IL6, and the activity of the nuclear factor kappa B (NF-κB): one of the transcription factors mediating the expression of proinflammatory genes [35]. Interestingly, APC also has neuroprotective effects. Using a model of hypoxia in human brain endothelial cells taken from a biopsy during brain surgery, it was shown that APC directly prevents apoptosis in hypoxic human brain endothelial cells through transcription-dependent inhibition of p53, normalization of the pro-apoptotic Bax/Bcl-2 ratio and reduction of caspase-3 signaling. The neuroprotective effect was confirmed in an in vivo focal ischemic stroke model in mice, with the administration of recombinant murine APC significantly reducing edema volume and infarction [36].

Moreover, K1 treatment was shown to reduce the expression of interleukin-6 (IL-6) mRNA in THP-1 cells (human macrophages) treated with LPS [33]. IL-6 has been implicated in the pathology of a wide range of diseases, including coronary artery disease (CAD) [37], which contributes to diabetes [38], aging [39,40] and cancer progression [41]. IL-6 activates major signaling pathways: the Janus-activated kinase-signal transducer and activator of transcription (JAK)-STAT) pathways, the Ras/Raf mitogen-activated protein kinase (MAPK, MEK/ERK) signaling cascade, and the phosphatidylinositol 3-kinase-dependent (PI3K/AKT) pathway [42,43,44]. The chemical structure of naphthoquinones implies that they have redox properties and can therefore exert anti-inflammatory properties. Vitamin K analogues inhibit LPS-induced IL-6 production, with menadione (K3) showing the strongest potential; in contrast, MK-4 or K1 must be metabolized to active forms to release their inhibitory potential [45]. Menadione is a product of phylloquinone metabolism during intestinal absorption [46,47,48].

## 3. Recommended Daily Intake (RDI) and Deficiency of Vitamin K

The recommended daily intake of vitamin K (all forms) ranges from 55 to 120 µg/day (Table 1); however, this value relates to the hepatic vitamin K requirement for the synthesis of blood clotting factors. The main storehouse of vitamin K is the liver, where most vitamin K absorbed from food resides; it only begins accumulating in remaining tissues when vitamin K is supplied at higher doses. The ratio of phylloquinone to menaquinone concentration varies considerably depending on the type of tissue, e.g., in the liver, the content of vitamin K1 is about 10 times higher than that of MK-4. High concentrations of phylloquinone are also observed in the heart muscle, while a high level of vitamin K2 is found in the pancreas, testes, and arterial walls [12,49]. It remains unclear whether this supply is sufficient to meet all physiological needs, especially health needs not related to clotting [18]. 

Currently, there is no fixed plasma/serum phylloquinone threshold indicating shortage or deficiency. Cohort studies performed by the Framingham Heart Study found that phylloquinone concentrations in plasma were approximately 1 nmol/L at a median consumption of vitamin K > 150 μg/d. Ingestion of ≤50 μg vitamin K resulted in 0.5 nmol/l phylloquinone in plasma [50]. In controlled nutritional studies, a reduced supply has been shown to soon result in a rapid decrease in the concentration of phylloquinone without disturbing coagulation processes; however, it is insufficient to support the maximum γ-carboxylation PIVKA-II in the liver or OC in the skeleton [51]. In addition, it is difficult to rebuild the correct levels using the recommended (90 µg/d) daily supply. Only an intake of two to five times greater concentrations allows the plasma phylloquinone concentration to be restored to the baseline [50,52].

**Table 1:** Recommended Daily Intake of Vitamin K for Adults.

	RDI (μg/day)	References
IOM ^1^ (2001)	90 μg/day women 120 μg/day men	[53]
WHO/FAO ^2^ (2004)	55 μg/day women 65 μg/day men	[54]
D-A-CH ^3^ (2015)	60 μg/day women 70 μg/day men	[54]
EFSA ^4^ Panel (2017)	70 μg/day for all adults	[54]
NIZP-PZH ^5^ (2020)	55 μg/day women 65 μg/day men	[55]

^1^ The United States Institute of Medicine; ^2^ The World Health Organization; ^3^ Deutsche Gesellschaft für Ernährung, Österreichische Gesellschaft für Ernährung, Schweizerische Gesellschaft für Ernährung; ^4^ European Food Safety Authority; ^5^ Narodowy Instytut Zdrowia Publicznego—Państwowy Zakład Higieny, Poland.

Although vitamin K deficiencies are uncommon in adults, they can be caused by malabsorption disorders, gastrointestinal disorders, antibiotic therapy, interactions with drugs, especially anticoagulants (VKA) used to reduce the risk of thromboembolic incidents or strokes in patients with cardiovascular diseases, and of course a diet with poor vitamin K content [56]. Poor diet still seems to be a problem depending on the region. The “National Fruit and Vegetable Consumption Survey”, carried out by Kantar Polska for the National Union of Fruit and Vegetable Producers Groups (the project was implemented in cooperation with the INSPIRE smarter branding agency), showed that in 2019, only 23% of respondents met the recommended daily intake of raw vegetables, and as many as 75% of Poles did not know how many fruit and vegetables they should eat. Only 15% of Poles ate properly when it comes to the amount of fruit and vegetables in the diet. Furthermore, the antibiotics mentioned above can increase the risk of hemorrhage, as shown in the population Based Nested Control-Case study [57] carried out on 6191 patients administered cephalosporin antibiotics, a group of broad-spectrum antibiotics often used in clinical and outpatient treatment. The study showed that the use of cephalosporin is associated with a 4.5-fold increase in the risk of bleeding. The study authors propose that a vitamin K deficit is caused by antibiotics eliminating vitamin K-producing intestinal bacteria, and by cephalosporin inhibiting the VK-dependent enzymes (VKOR and GGCX). In addition, the authors observed that poor nutrition was associated with an increase in bleeding risk by 40%. 

The supply of vitamin K differs depending on the region of the world and is directly related to the amount of vegetables in the diet [2,12,58]. The Dutch population is characterized by a high supply of vitamin K (K1: 124–375 µg per day; K2 between 10 µg and 45 µg per day), resulting from high consumption of vegetables and dairy products [59]. Asian populations demonstrate significantly higher vitamin K consumption compared to the British population; this indicates a link between greater vitamin K supply and increased consumption of vegetables, algae, and soybean fermentation products, typical of diets in the Far East, which significantly distinguishes it from the Western diet [58,60].

## 4. Anti-Cancer Properties

Natural forms of vitamin K–K1 and K2—have only a low potential for toxicity. Therefore, the National Academy of Sciences and the European Food Safety Authority (EFSA) have not set an upper limit for vitamin K, which indicates that even a high supply will not cause any harmful effects to most people. However, K3 may demonstrate harmful potential: synthetic vitamin K3 can lead to liver damage and destruction of oxygen-carrying red blood cells [61,62]. Nevertheless, despite its harmful effect on humans, vitamin K3 has shown anti-cancer and anti-inflammatory properties in numerous studies. This may indicate that vitamin K has an important preventive role, especially K1, which is converted into menadione in the human intestinal cells [63], in the diet.

### 4.1. DNA Damage

Menadione, a product of phylloquinone metabolism, can participate in redox reactions and generates reactive oxygen species (ROS), which then leads to damage to DNA and other macromolecules. ROS production plays a primal and important role in menadione-mediated DNA damage. Quinones generate semiquinones by single-electron reactions or hydroquinones in the two-electron reaction [64]. The semiquinone (SQ) is a partially reduced free radical form (Q•^−^) that can be re-oxidized by molecular oxygen (O_2_ to generate O^2−^), so semiquinones can effectively contribute to the formation of superoxides in biological systems [65]. Like another quinone, doxorubicin, menadione exerts its cytotoxic effects by stimulating the generation of oxidative stress, leading to DNA damage [66].

Ngo et al. showed that when applied in cytotoxic doses, menadione induces the formation of ssDNA (SSB) and dsDNA (DSB) breaks in breast cancer cells (MCF-7) [66]. Researchers have shown that the number of DNA breaks is directly proportional to the concentration of menadione and is directly related to the level of ROS. Similar results were noted for the K562 line of chronic myeloid leukemia, where low concentrations of menadione resulted in an increase in the number of SSBs [67]. DNA breaks appeared as early as five minutes after exposure of cells to 15 µM menadione. Interestingly, damage repair was more impaired after incubating cells with menadione than with another quinone (2,3-dimethyl-1,4-naphthoquinone). The authors suggest that quinones induce cytostasis while maintaining the integrity of cell membranes. Low, non-toxic concentrations of menadione can generate DNA-damaging ROS in close proximity to DNA [68] in the presence of Fe^2+^; ssDNA breaks are a signal that activates the poly ADP ribose polymerase (PARP1) [69,70], which consumes the cellular NAD^+^ pool and activates the BER DNA repair process [67]. This results in metabolic exhaustion and a significant inhibition of cell proliferation.

**Figure 3:**
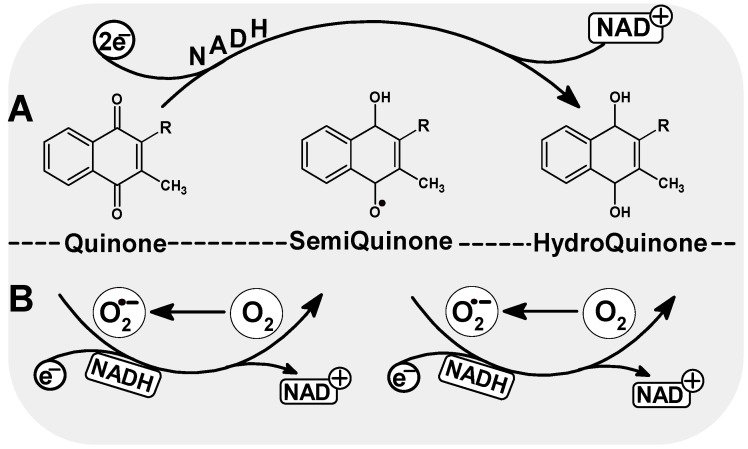
Possible quinone reduction reactions. A. two-electron reduction, consisting of a direct, one-step reduction to hydroquinone; this process does not generate ROS; B. single-electron reduction to form an unstable semiquinone radical. In the presence of molecular oxygen, ROS (O_2_^−^) can be formed in the reoxidation reaction.

ROS and the semiquinone radical generated during the one-electron reduction of menadione are reported as the major contributors to the DNA damage resulting from exposure to menadione (Figure 3). The intercalating mechanism of covalent binding of the semichinone radical to DNA was demonstrated after radioactive menadione incubation with DNA and measurements of covalently bound activity [64]. Intercalation to DNA by covalent binding of the semiquinone radical to nucleic acid has been observed for other quinone anti-cancer drugs such as adriamycin or daunorubicin [71]. Therefore, the ability of adriamycin to induce extensive chromosome aberration and increase the incidence of sister chromatid exchange is explained by its strong covalent bond to DNA. The semiquinone radical may take part in the formation of DNA strand breaks, although it is more often the result of the ROS generated in the one-electron reduction of menadione. A strong electrophilic radical initiates strand breakage through an electrophilic attack on the carbons of the deoxyribose backbone [72]. Menadione has been shown to induce DNA strand breaks in various cell types [73,74,75,76,77]. Free radicals resulting from one-electron reduction of menadione (Figure 3) are also responsible for the depletion of GSH—preventing DNA binding of reactive metabolites of menadione [64].

Later studies on cardiomyocytes by Loor et al. confirmed the potential of menadione to produce rapid and significant oxidative stress in various subcellular compartments [69]. After an incubation of 25 min, menadione (25 µM) caused oxidation of RoGFP (the radiometric redox sensor), reaching 75% in the cytosol and 85% in the mitochondrial matrix; compared to the initial—reducing conditions, in the cytosol, RoGFP oxidation averaged from 20 to 30%. Moreover, during a four-hour incubation period, a decrease in the mitochondrial membrane potential was observed, reaching 39% of the initial value. Studies on PARP-1 -/- murine embryos confirmed that PARP1 activation is an important component of the menadione-induced death pathway. Subsequent tests conducted on the Fuchs’ corneal dystrophy (FECD) using menadione as oxidative stress in normal endothelial cell lines (HCERC-21T and HCECE) showed that menadione damages not only nuclear DNA (nDNA), but also mitochondrial DNA (mtDNA) [78]. Menadione induces various dysfunctions in MT, such as depolarization of the MT membrane, a change in organelle morphology, and at higher concentrations (50 and 100 μM) ATP depletion. After a six-hour incubation period, the release of cytochrome C into the cytosol and the cutting of procaspase nine and three were observed. These processes are accompanied by an increase in Ca^2+^ concentration in the cellular cytosol [79], which is an important secondary messenger in controlling cell death [80,81]. Menadione undergoes redox cycles, induces ROS generation, and repairs DNA without showing any carcinogenic potential [82]. In pancreatic acinar cells, it was observed that the application of 30 μM menadione triggered a marked and significant increase in ROS levels immediately after exposure to menadione [83]. Menadione generates acute ROS production and promotes the opening of permeability transition pores, and induces Ca^2+^ fluctuations in pancreatic acinar cells, thus promoting programmed cell death [79,83]. A similar mechanism of action is demonstrated by K2: treatment of K2 human ovarian cancer cells results in the production of peroxides and H_2_O_2_ and induction of apoptosis resulting from K2-mediated depolarization of mitochondrial membranes [84].

Highly mutagenic DNA damage is 8-oxo-2-deoxyguanosine (8-oxo-dG) considered as a marker of oxidative stress. 8-oxo-dG can mismatch with dA generating a G to T transversion. In the presence of ROS, guanine (G) is easily oxidized, making 8-oxo-2-deoxyguanine (8-oxoGua) the most abundant DNA damage [85,86,87,88,89,90]. In a study carried out on rat hepatocytes exposed to menadione, DNA breaks were observed, although no significant elevated levels of 8-oxo-dG were observed after the administration of 100 µM menadione (after 15 and 90 min) [91]. In other studies, using electron spin resonance (ESR) analysis, it was found that 100 µM menadione induced a significant increase in the amount of 8-oxo-dG in Caco-2 cells after two hours’ incubation [92]. Similarly, the incubation of human breast carcinoma cells in the presence of 25 or 50 μM menadione for one hour generated 8-oxo-dG in mtDNA, and higher concentrations (100 and 200 μM) resulted in DNA strand breakage [93]. Analysis of gene expression as a result of treatment of Caco-2 menadion cells found 979 genes to demonstrate altered expression, and menadione modulated a number of significant processes including the transcription and regulation of the cell cycle, glutathione metabolism, cytoskeleton remodeling or WNT signaling [91]

### 4.2. Anti-cancer Effect

The anti-cancer potential of vitamin K has been the subject of research for several decades; its findings indicate that vitamin K (especially K2 and K3) can inhibit various types of cancer cells (ovarian cancer, leukemia, bladder cancer, hepatocellular carcinoma) through the induction of apoptosis or autophagy. The quinone moiety is present in the chemical structure of many cancer chemotherapeutic drugs such as anthracyclines daunorubicin, doxorubicin, mitoxantrone, or banoxantrone [94,95,96,97,98].

Various studies suggest different scenarios for the activity of vitamin K as an anticancer agent. In one study provided by Hitomi et al., treatment with vitamin K2 or K3 (400 µM) reduced the growth of hepatocellular carcinoma cells (HCC) implanted subcutaneously in mice [99]. An immunohistochemical evaluation of the tumors 53 days after inoculation revealed significant suppression of cyclin D1 and cyclin-dependent kinase 4 (CDK4) protein expression, and induced G1 cell cycle arrest and tumor growth inhibition. Subsequently, studies by Ozaki et al. confirmed that vitamin K2 inhibits the activity of the cyclin D1 promoter in an NFκB-dependent manner [100]. K2 has also been determined to inhibit IκB kinase (IKK) activity, thereby inhibiting the phosphorylation of IκBα (an inhibitor of NF-κB), thus preventing NF-κB activation and nuclear translocation (NF-κB is associated with cell growth and carcinogenesis) and cell cycle inhibition. Another study investigated the regulation of hepatoma-derived growth factor (HDGF) by K2 in hepatocellular carcinoma cells. It has been observed that vitamin K can also have an inhibitory effect on HCC cells by suppressing HDGF, as it significantly decreases protein expression [101]. Vitamin K2 can also induce cancer cell differentiation by modulating connexin gene expression [102]. Connexin (Cx) mediates intercellular communication that maintains tissue homeostasis, as the components of gap-junction connections ensure proper intercellular interactions, which are disturbed in neoplasms [103]. K2 can alter the expression pattern of connexins at the transcription level: it enhances the expression of Cx32 and suppresses Cx43, which inhibits proliferation and induces a normal liver phenotype.

K2 has been found to induce apoptosis in cancer cells. The use of K2 (30 µM) in the human promyelocytic cell line HL60 leads to changes in mitochondrial membrane potential, the release of cytochrome C, and induction of the apoptosis pathway mediated by the activation of Bak [Bcl-2 antagonist killer 1] [104]. Other data show that vitamin K may limit the survival of some pancreatic cancer lines (MiaPaCa2 and PL5). Its IC50 value was estimated to be 150 µM and 75 µM for K1 and K2, respectively. Inhibition of cell survival is mediated through apoptosis in the MAP kinase pathway. Vitamin K1 and K2 induce ERK phosphorylation in a time- and dose-dependent manner [105].

Vitamin K is also believed to exert anti-cancer activities by inducing autophagy. Depending on the level of cellular expression of Bcl-2, K2 may induce apoptosis or, in the case of Bcl-2 overexpression, direct the cell to autophagy [106]. In bladder cancer cells (T24), vitamin K2 significantly induces PI3K/Akt phosphorylation and increases expression of HIF-1α, intensifying glucose consumption and lactate formation. In response to the metabolic stress that arises, K2 triggers AMPK-dependent autophagic cell death [107]. Interestingly, a synthetic form of vitamin K, chloro-derivative menadione (VKT-2), also shows similar activity, inducing apoptosis and autophagy, and inhibits cell migration in liver tumor cells (HUH-7) [108]. In addition, the molecular goal of the analogue used was histone deacetylase (HDACS6) epigenetic regulatory protein. HDAC6 deacetylates not only histones but also cytoplasmic proteins such as α-tubulin, HSP90, p53, and cortactin. Using thermophoresis in microscales and enzymatic tests, VKT-2 was found to bind to HDAC6 and inhibit its activity resulting in hyperacetylation of α-tubulin, stabilization of microtubules and suppression of cell mobility. This suggests that vitamin K could be used in an epigenetic anti-cancer therapeutic strategy.

Another molecular target for vitamin K appears to be γ DNA polymerase. γ DNA polymerase maintains the mtDNA genome and its replication, and is responsible for all aspects of mitochondrial DNA from synthesis to repair. Mutsubara et al. showed the inhibitory effect of menadione on γ DNA polymerase by DNA polymerase tests [109]. Menadione selectively and dose-dependently inhibited the enzyme activity, reaching 50% inhibition at 6 µM menadione without affecting other polymerases. Interestingly, quinones with long side chains did not show the same activity as menadione. The authors postulate that the inhibitory effect of K3 is achieved through interaction with DNA as a template-DNA primer and inhibition of enzyme activity through direct interaction with γ DNA polymerase. This activity is related to the anti-angiogenic effect of menadione examined in an ex vivo and in vivo angiogenesis assay and indicates the function of K3 as an anti-tumor agent.

An interesting aspect of the K vitamins is their synergistic action with other compounds demonstrating anti-cancer activity. This is important in the context of combination therapy, which is a frequently used strategy in cancer treatment [110]. The combination of two or more drugs may lead to increased efficacy, multidirectional cellular activity, or the suppression of drug resistance, thus permitting lower doses. Greater benefits can be achieved if one of the combined agents is a micronutrient beneficial to health. Numerous studies indicate that the K vitamins have an additive or synergistic effect on various chemotherapeutic agents. Menadione has been found to be effective against multi-drug resistance in cancer cells by an additive effect with Mercaptopurine, Cytarabine, Hydroxyurea, VP-16, Vincristine, Doxorubicin, Mitoxantrone, or Mitomycin C, and synergistic effects with Fluorouracil, Cis-platin, and Dacarbazine. It only demonstrates an antagonistic effect when paired with methotrexate [111].

A strong synergism between K1 and sorafenib has been demonstrated in numerous studies [112,113,114,115]. Sorafenib (SFB), intended for the treatment of advanced kidney cancer and hepatocellular carcinoma, is an inhibitor of many kinases, both serine-threonine (RAF) and receptor tyrosine kinases (eg. ERK). It was observed that in HCC cells, K1 strongly enhances the sorafenib-induced inhibition of tumor cell proliferation [116]. Combined K1/SFB treatment allowed up to a 6.7-fold reduction in the concentration of sorafenib, with tumor reduction accompanied by reduced phosphorylation and inhibition of the RAF/MAPKK/ERK pathway. A similar observation was noted for pancreatic cancer cells when K1/SFB (50 µM/2.5 µM) was used, with a significant reduction in ERK and MEK phosphorylation, induction of apoptosis, and thus tumor regression [117].

K2 also has a chemosensitizing effect on cancer cells, with increased therapeutic benefits associated with SFB, 5-FU, or retinoid treatment [118,119,120]. The use of retinoids (analogs of vitamin A) and K2 resulted in synergistic inhibition of the Ras/ERK pathway in HCC and leukemia cells, triggered dephosphorylation of retinoid X receptors (RXR) and enabled their stimulation with retinoids [121], thus increasing apoptosis. Importantly, the K2/retinoid combination does not impair the growth and survival of normal hepatocytes. Interestingly, another micronutrient, tocopherol, can antagonize the anti-apoptotic effect of K2. As a strong antioxidant, tocopherol counteracted K2-mediated peroxide production and inhibited mitochondrial membrane polarization, which limited apoptosis in ovarian cancer cells [84].

In contrast to tocopherol, another micronutrient, ascorbic acid (AA), has a synergistic effect on K3 [73,122,123]. The AA/K3 association leads to an excessive increase in oxidative stress and a decrease in the potential of the mitochondrial membrane, which is a crucial trigger of tumor cell death [122,124,125]. This pairing enhances the effect of numerous chemotherapeutic agents. When tested with 13 chemotherapeutic agents (ABT-737, barasertib, bleomycin, BEZ-235, bortezomib, cisplatin, everolimus, lomustine, lonafarnib, MG-132, MLN-2238, palbociclib, and PI-103) AA/K3 demonstrated highly specific synergistic suppression of cancer cell growth at pharmacological concentrations; it produces metabolic changes that promote increased sensitivity of cancer cells to conventional anti-cancer therapy, while not adversely affecting normal cell survival [126].

According to the results of in vitro studies on cells, the data from a prospective EPIC-Heidelberg (European Prospective Investigation into Cancer and Nutrition–Heidelberg) cohort study, although not statistically significant, indicate that K2 treatment may have a positive effect on the therapy of cancer patients, reduce the risk of developing cancer, and lower mortality [127]. In a prospective study of 101,695 American adults, those with higher intakes of phylloquinone but not menaquinones had a lower risk of pancreatic cancer [128]. It has been suggested that increasing consumption of phylloquinone-rich foods may be an effective strategy for preventing pancreatic cancer. A study of the effect of the K2 analog menatetrenone on the recurrence rate of hepatocellular carcinoma (HCC) suggests that it may improve survival among patients treated surgically. Sixty patients were divided into two groups, one given a placebo and the other treated with 45 mg menatetrenone; it was found that menatetrenone significantly reduced the cumulative HCC recurrence rate (*p* = 0.0002) [129]. A meta-analysis of randomized control trials (RCT) and cohort studies on the effectiveness of K2 therapy in HCC patients showed that K2 can reduce the frequency of relapses and improve overall survival in HCC patients as early as one year after surgery [130].

## 5. Summary

One of the major determinants of vitamin K status is dietary vitamin K intake, and this varies widely within age groups and population subgroups. Similarly, due to the wide range of biochemical markers used in studies, the vitamin K status of different populations varies greatly. Measurement of the plasma vitamin K level does not provide accurate and authoritative information, as the presence of carboxylated proteins in serum does not necessarily indicate the normal state of vitamin K in other organs, and plasma phylloquinone concentrations only reflect consumption for the past 24 h [131]. Nevertheless, a vitamin K1-deficient diet results in functional subclinical vitamin K deficiency without disturbing blood clotting [132].

There is no doubt that vitamin K should be recognized as more than just a micronutrient related to regulating coagulation, and rather as a broadly pleiotropic factor in cell metabolism. Vitamin K is involved in many cellular processes, as a factor necessary for the γ-glutamyl carboxylation of all vitamin K dependent proteins, making it a vital component in clotting, bone formation, and the prevention of vascular calcification. Many observations attribute the potential of vitamin K to prevent the development of certain pathological conditions to its anti-inflammatory effects. In addition, recent results indicate that poor vitamin K status is associated with the more serious outcomes of COVID-19, again related to the anti-inflammatory activity of vitamin K [133]. This anti-inflammatory effect can also influence carcinogenesis.

Finally, vitamin K has been found to affect the expression of proteins and genes. By modulating the signaling cascade, it can also inhibit the cell cycle or induce apoptosis, thus potentially exerting anti-cancer potential. Therefore, ensuring adequate vitamin K intake by maintaining a healthy diet including foods rich in vitamin K can help reduce incidences of cancer/mortality and increase longevity.

While new information about the involvement of vitamin K in cell and organism metabolism continues to emerge, it is easy to get lost in the whole wealth of available information and overlook essential, crucial, or groundbreaking information. There is a need not only for in-depth research on how vitamin K is involved in metabolic pathways, but also for its interaction with, e.g., other vitamins. Vitamins are classified as dietary supplements, not drugs; nevertheless all vitamins have significant physiological and pharmacological effects and therefore may interact with drugs or other supplements. There is a need for systematic reviews and meta-analyses to gain a greater sense of understanding and a clear picture of vitamin K’s response to human health.
